# Writing a scientific paper: Where to start from?

**DOI:** 10.1590/2176-9451.19.1.001-001.edt

**Published:** 2014

**Authors:** David Normando

**Affiliations:** editor-in-chief.

"Our end was just the beginning." (Yuri Popoff, bassist and composer from Minas Gerais)

It is not our aim, in the next few lines, to make our reader become a contumacious scientific writer. Scientific writing requires practice and there are only a few courses that can guide us to a shortcut. However, there are some useful pieces of advice that I pass on based on the experience I have had as a reviewer, editor and, especially, as an author.

Good scientific research begins with an original idea that is put into practice by means of a robust methodology. If there is a good question, the answer will come in a more interesting way. Once you reliably find the answer, you will only need a low dose of efficient communication. I have come across excellent papers that get lost due to poor writing. However, the opposite is more common. The board of editors will be more interested in correcting minor writing mistakes.

Where to start from? How about the end? Exactly. Start with the conclusion. To my view, one of the most common mistakes is to start writing by the introduction. How can we present something if we do not know what it is? This ordinary path, in my opinion, results from the attempt to make the best of the project that originated the study we write about. As a consequence, we end up with a long introduction that is far from conveying the main message of the research: the conclusion. Thus, starting with the conclusion infuses our mind with the draft of the path that we would like to reveal to our readers.

Afterwards, it is important to keep the conclusion in mind to write the paper in a brief and linear manner. Another common mistake in scientific writing is to be prolix, write more than necessary, especially in the introduction. The researcher intensely lives what he does, sleeping and dreaming about the object of his experiment or observation. And through inebriant osmosis, he starts to believe that everything he writes is utterly important. However, it is worth that the author put himself in the reader's shoes. In this case, less is more. We live in the age of - fast - communication. Short texts and the right words are more appropriate. For this reason, simply read and reread what you have written, and, without fear or guilt, cut everything you think will hinder the comprehension of the text. Many scientific journals of great impact, such as Nature and Science, require that the authors submit much shorter texts than we are used to in Dentistry field. There is a good reason for that.

Draft. That's it. Draw up a strategic draft, a sketch of all the information you would like to present and define where every item will be within the structure of the text. Remember to keep the conclusion in mind. As you find new interesting information, first ask yourself whether this knowledge is really important for the comprehension of the text. Only after you find an affirmative convincing answer, point out where this questioning will be in the structure of the text.

Within the IMRaD structure (Introduction, Methodology, Results and Discussion), after the conclusion, I usually move forward to the results, followed by the discussion and the material and methods. The introduction is the last part. In all of these parts, do not hesitate to cut the excesses. Researchers usually find it difficult to discard data that did not produce any useful information in their experiment. Similarly, they find it difficult to understand the reason why we have to take a step back in order to search for new information that may be key to the success of the study. Eliminating excessive data greatly facilitates the process of writing a manuscript.

Leave the title to the end. You may even write a temporary title, however, when the text is ready, analyze if the title is suitable to communicate its content, especially what is in the conclusion. We have to understand that the title is the abstract of the abstract, and that is the reason why it should not be too long. Long titles, as well as long texts, instigate readers' disinterest. The title does not have to necessarily communicate everything.

Reread the title of this editorial. All right, this is not a research. An editorial is freer to flow. But analyze it. The text does not reflect on how to start writing a manuscript, only. There are other pieces of information. Should I have mentioned all pieces of information in the title, our beginning would have reached its end right there.

